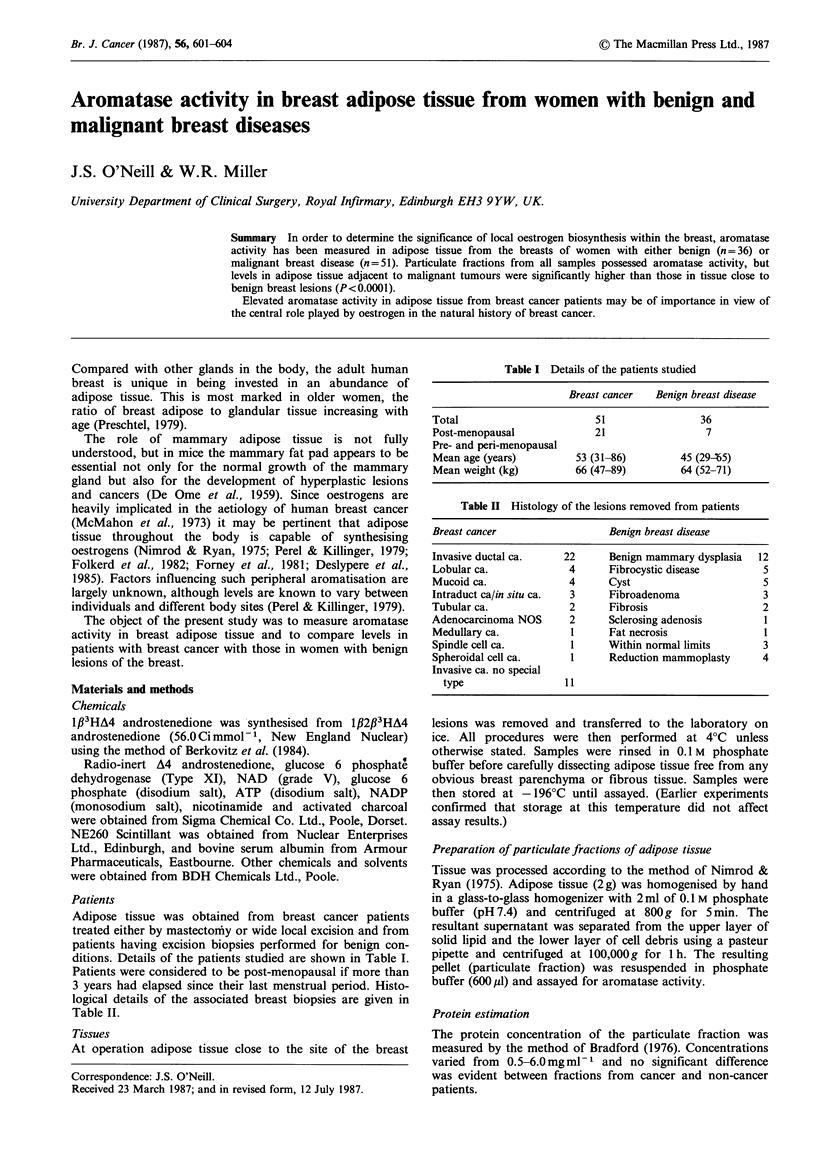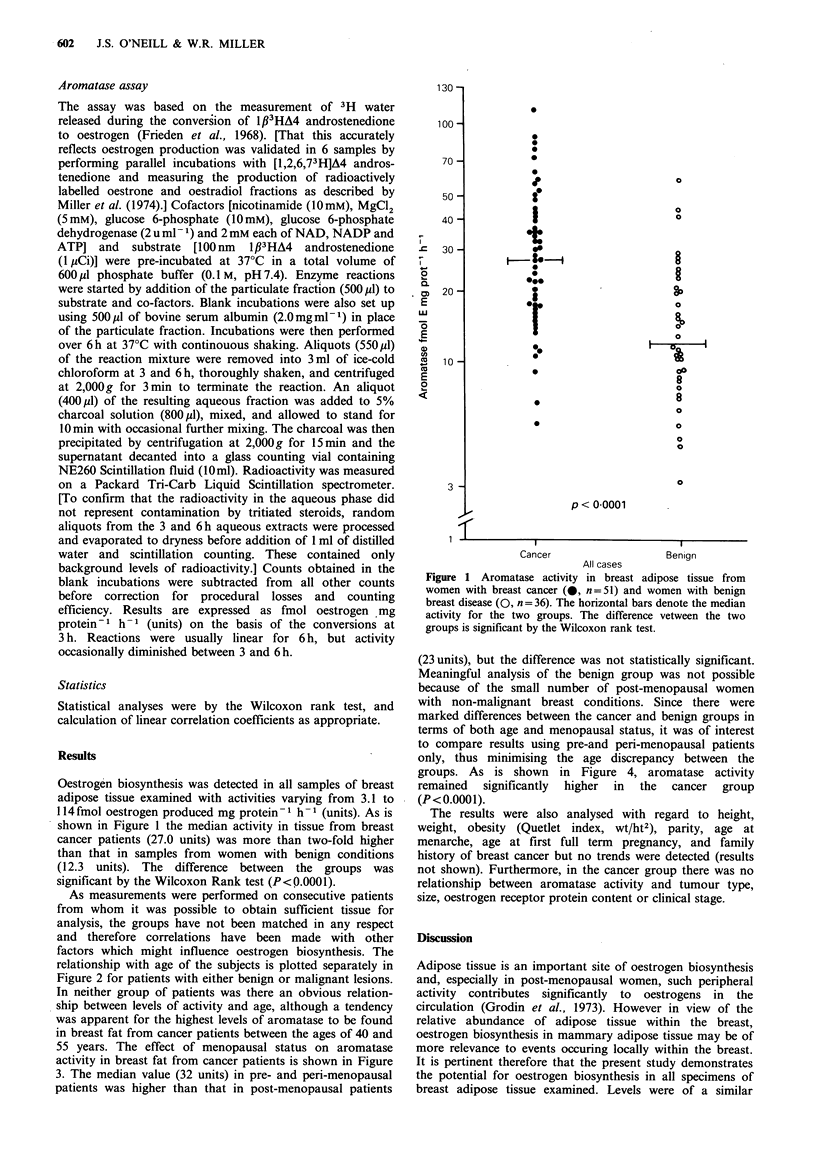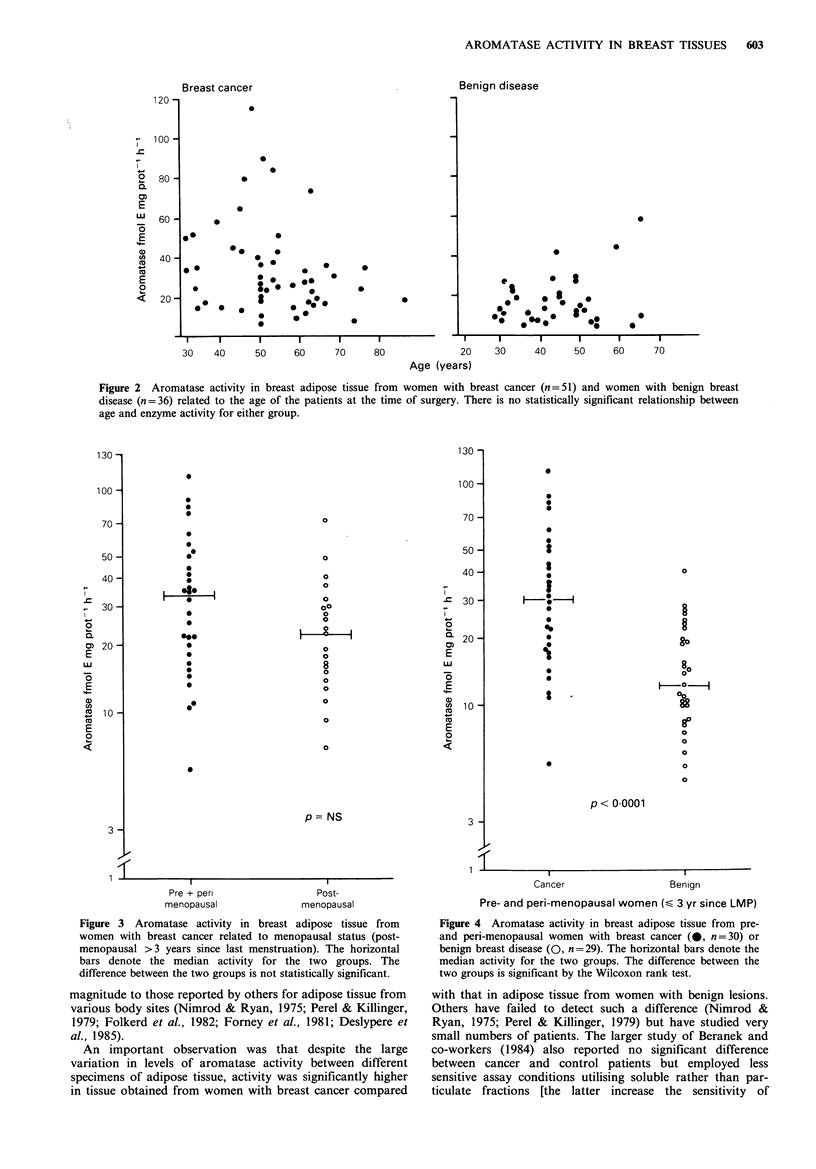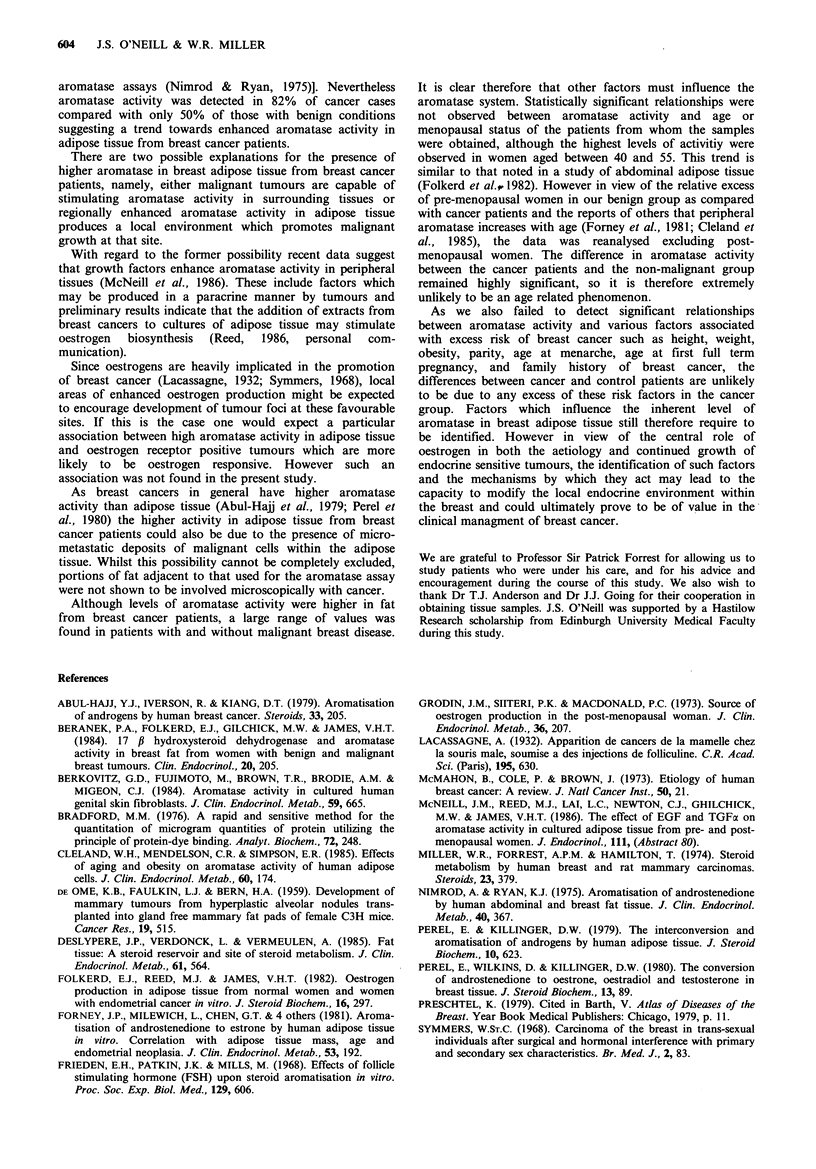# Aromatase activity in breast adipose tissue from women with benign and malignant breast diseases.

**DOI:** 10.1038/bjc.1987.248

**Published:** 1987-11

**Authors:** J. S. O'Neill, W. R. Miller

**Affiliations:** University Department of Clinical Surgery, Royal Infirmary, Edinburgh, UK.

## Abstract

In order to determine the significance of local oestrogen biosynthesis within the breast, aromatase activity has been measured in adipose tissue from the breasts of women with either benign (n = 36) or malignant breast disease (n = 51). Particulate fractions from all samples possessed aromatase activity, but levels in adipose tissue adjacent to malignant tumours were significantly higher than those in tissue close to benign breast lesions (P less than 0.0001). Elevated aromatase activity in adipose tissue from breast cancer patients may be of importance in view of the central role played by oestrogen in the natural history of breast cancer.


### Aromatase activity in breast adipose tissue from women with benign and malignant breast diseases

J.S. O'Neill & W.R. Miller

*University Department of Clinical Surgery, Royal Infirmary, Edinburgh EH3 9YW, UK.*

**Summary** In order to determine the significance of local oestrogen biosynthesis within the breast, aromatase activity has been measured in adipose tissue from the breasts of women with either benign ($n=36$) or malignant breast disease ($n=51$). Particulate fractions from all samples possessed aromatase activity, but levels in adipose tissue adjacent to malignant tumours were significantly higher than those in tissue close to benign breast lesions ($P<0.0001$).

Elevated aromatase activity in adipose tissue from breast cancer patients may be of importance in view of the central role played by oestrogen in the natural history of breast cancer.

Compared with other glands in the body, the adult human breast is unique in being invested in an abundance of adipose tissue. This is most marked in older women, the ratio of breast adipose to glandular tissue increasing with age (Preschtel, 1979).

The role of mammary adipose tissue is not fully understood, but in mice the mammary fat pad appears to be essential not only for the normal growth of the mammary gland but also for the development of hyperplastic lesions and cancers (De Ome *et al.*, 1959). Since oestrogens are heavily implicated in the aetiology of human breast cancer (McMahon *et al.*, 1973) it may be pertinent that adipose tissue throughout the body is capable of synthesising oestrogens (Nimrod & Ryan, 1975; Perel & Killinger, 1979; Folkerd *et al.*, 1982; Forney *et al.*, 1981; Deslypere *et al.*, 1985). Factors influencing such peripheral aromatisation are largely unknown, although levels are known to vary between individuals and different body sites (Perel & Killinger, 1979).

The object of the present study was to measure aromatase activity in breast adipose tissue and to compare levels in patients with breast cancer with those in women with benign lesions of the breast.

### Materials and methods

### *Chemicals*

$1\beta^3H\Delta4$ androstenedione was synthesised from $1\beta2\beta^3H\Delta4$ androstenedione ($56.0\,Ci\,mmol^{-1}$, New England Nuclear) using the method of Berkovitz *et al.* (1984).

Radio-inert Δ4 androstenedione, glucose 6 phosphate dehydrogenase (Type XI), NAD (grade V), glucose 6 phosphate (disodium salt), ATP (disodium salt), NADP (monosodium salt), nicotinamide and activated charcoal were obtained from Sigma Chemical Co. Ltd., Poole, Dorset. NE260 Scintillant was obtained from Nuclear Enterprises Ltd., Edinburgh, and bovine serum albumin from Armour Pharmaceuticals, Eastbourne. Other chemicals and solvents were obtained from BDH Chemicals Ltd., Poole.

### *Patients*

Adipose tissue was obtained from breast cancer patients treated either by mastectomy or wide local excision and from patients having excision biopsies performed for benign conditions. Details of the patients studied are shown in Table I. Patients were considered to be post-menopausal if more than 3 years had elapsed since their last menstrual period. Histological details of the associated breast biopsies are given in Table II.

### *Tissues*

At operation adipose tissue close to the site of the breast lesions was removed and transferred to the laboratory on ice. All procedures were then performed at 4°C unless otherwise stated. Samples were rinsed in 0.1 M phosphate buffer before carefully dissecting adipose tissue free from any obvious breast parenchyma or fibrous tissue. Samples were then stored at −196°C until assayed. (Earlier experiments confirmed that storage at this temperature did not affect assay results.)

**Table I** Details of the patients studied

| | *Breast cancer* | *Benign breast disease* |
|---|---|---|
| Total | 51 | 36 |
| Post-menopausal | 21 | 7 |
| Pre- and peri-menopausal | | |
| Mean age (years) | 53 (31–86) | 45 (29–65) |
| Mean weight (kg) | 66 (47–89) | 64 (52–71) |

**Table II** Histology of the lesions removed from patients

| *Breast cancer* | | *Benign breast disease* | |
|---|---|---|---|
| Invasive ductal ca. | 22 | Benign mammary dysplasia | 12 |
| Lobular ca. | 4 | Fibrocystic disease | 5 |
| Mucoid ca. | 4 | Cyst | 5 |
| Intraduct ca/*in situ* ca. | 3 | Fibroadenoma | 3 |
| Tubular ca. | 2 | Fibrosis | 2 |
| Adenocarcinoma NOS | 2 | Sclerosing adenosis | 1 |
| Medullary ca. | 1 | Fat necrosis | 1 |
| Spindle cell ca. | 1 | Within normal limits | 3 |
| Spheroidal cell ca. | 1 | Reduction mammoplasty | 4 |
| Invasive ca. no special type | 11 | | |

### *Preparation of particulate fractions of adipose tissue*

Tissue was processed according to the method of Nimrod & Ryan (1975). Adipose tissue (2 g) was homogenised by hand in a glass-to-glass homogenizer with 2 ml of 0.1 M phosphate buffer (pH 7.4) and centrifuged at 800 *g* for 5 min. The resultant supernatant was separated from the upper layer of solid lipid and the lower layer of cell debris using a pasteur pipette and centrifuged at 100,000 *g* for 1 h. The resulting pellet (particulate fraction) was resuspended in phosphate buffer (600 μl) and assayed for aromatase activity.

### *Protein estimation*

The protein concentration of the particulate fraction was measured by the method of Bradford (1976). Concentrations varied from $0.5–6.0\,mg\,ml^{-1}$ and no significant difference was evident between fractions from cancer and non-cancer patients.

Correspondence: J.S. O'Neill.
Received 23 March 1987; and in revised form, 12 July 1987.

*Aromatase assay*

The assay was based on the measurement of $^3$H water released during the conversion of 1$\beta^3$HΔ4 androstenedione to oestrogen (Frieden *et al.*, 1968). [That this accurately reflects oestrogen production was validated in 6 samples by performing parallel incubations with [1,2,6,7$^3$H]Δ4 androstenedione and measuring the production of radioactively labelled oestrone and oestradiol fractions as described by Miller *et al.* (1974).] Cofactors [nicotinamide (10 mM), $MgCl_2$ (5 mM), glucose 6-phosphate (10 mM), glucose 6-phosphate dehydrogenase (2 u ml$^{-1}$) and 2 mM each of NAD, NADP and ATP] and substrate [100 nm 1$\beta^3$HΔ4 androstenedione (1 $\mu$Ci)] were pre-incubated at 37°C in a total volume of 600 $\mu$l phosphate buffer (0.1 M, pH 7.4). Enzyme reactions were started by addition of the particulate fraction (500 $\mu$l) to substrate and co-factors. Blank incubations were also set up using 500 $\mu$l of bovine serum albumin (2.0 mg ml$^{-1}$) in place of the particulate fraction. Incubations were then performed over 6 h at 37°C with continouous shaking. Aliquots (550 $\mu$l) of the reaction mixture were removed into 3 ml of ice-cold chloroform at 3 and 6 h, thoroughly shaken, and centrifuged at 2,000 *g* for 3 min to terminate the reaction. An aliquot (400 $\mu$l) of the resulting aqueous fraction was added to 5% charcoal solution (800 $\mu$l), mixed, and allowed to stand for 10 min with occasional further mixing. The charcoal was then precipitated by centrifugation at 2,000 *g* for 15 min and the supernatant decanted into a glass counting vial containing NE260 Scintillation fluid (10 ml). Radioactivity was measured on a Packard Tri-Carb Liquid Scintillation spectrometer. [To confirm that the radioactivity in the aqueous phase did not represent contamination by tritiated steroids, random aliquots from the 3 and 6 h aqueous extracts were processed and evaporated to dryness before addition of 1 ml of distilled water and scintillation counting. These contained only background levels of radioactivity.] Counts obtained in the blank incubations were subtracted from all other counts before correction for procedural losses and counting efficiency. Results are expressed as fmol oestrogen mg protein$^{-1}$ h$^{-1}$ (units) on the basis of the conversions at 3 h. Reactions were usually linear for 6 h, but activity occasionally diminished between 3 and 6 h.

*Statistics*

Statistical analyses were by the Wilcoxon rank test, and calculation of linear correlation coefficients as appropriate.

### Results

Oestrogen biosynthesis was detected in all samples of breast adipose tissue examined with activities varying from 3.1 to 114 fmol oestrogen produced mg protein$^{-1}$ h$^{-1}$ (units). As is shown in Figure 1 the median activity in tissue from breast cancer patients (27.0 units) was more than two-fold higher than that in samples from women with benign conditions (12.3 units). The difference between the groups was significant by the Wilcoxon Rank test ($P<0.0001$).

As measurements were performed on consecutive patients from whom it was possible to obtain sufficient tissue for analysis, the groups have not been matched in any respect and therefore correlations have been made with other factors which might influence oestrogen biosynthesis. The relationship with age of the subjects is plotted separately in Figure 2 for patients with either benign or malignant lesions. In neither group of patients was there an obvious relationship between levels of activity and age, although a tendency was apparent for the highest levels of aromatase to be found in breast fat from cancer patients between the ages of 40 and 55 years. The effect of menopausal status on aromatase activity in breast fat from cancer patients is shown in Figure 3. The median value (32 units) in pre- and peri-menopausal patients was higher than that in post-menopausal patients

**Figure 1** Aromatase activity in breast adipose tissue from women with breast cancer (●, $n=51$) and women with benign breast disease (○, $n=36$). The horizontal bars denote the median activity for the two groups. The difference vetween the two groups is significant by the Wilcoxon rank test.

(23 units), but the difference was not statistically significant. Meaningful analysis of the benign group was not possible because of the small number of post-menopausal women with non-malignant breast conditions. Since there were marked differences between the cancer and benign groups in terms of both age and menopausal status, it was of interest to compare results using pre-and peri-menopausal patients only, thus minimising the age discrepancy between the groups. As is shown in Figure 4, aromatase activity remained significantly higher in the cancer group ($P<0.0001$).

The results were also analysed with regard to height, weight, obesity (Quetlet index, wt/ht$^2$), parity, age at menarche, age at first full term pregnancy, and family history of breast cancer but no trends were detected (results not shown). Furthermore, in the cancer group there was no relationship between aromatase activity and tumour type, size, oestrogen receptor protein content or clinical stage.

### Discussion

Adipose tissue is an important site of oestrogen biosynthesis and, especially in post-menopausal women, such peripheral activity contributes significantly to oestrogens in the circulation (Grodin *et al.*, 1973). However in view of the relative abundance of adipose tissue within the breast, oestrogen biosynthesis in mammary adipose tissue may be of more relevance to events occuring locally within the breast. It is pertinent therefore that the present study demonstrates the potential for oestrogen biosynthesis in all specimens of breast adipose tissue examined. Levels were of a similar

**Figure 2** Aromatase activity in breast adipose tissue from women with breast cancer ($n=51$) and women with benign breast disease ($n=36$) related to the age of the patients at the time of surgery. There is no statistically significant relationship between age and enzyme activity for either group.

**Figure 3** Aromatase activity in breast adipose tissue from women with breast cancer related to menopausal status (post-menopausal >3 years since last menstruation). The horizontal bars denote the median activity for the two groups. The difference between the two groups is not statistically significant.

**Figure 4** Aromatase activity in breast adipose tissue from pre- and peri-menopausal women with breast cancer (●, $n=30$) or benign breast disease (○, $n=29$). The horizontal bars denote the median activity for the two groups. The difference between the two groups is significant by the Wilcoxon rank test.

magnitude to those reported by others for adipose tissue from various body sites (Nimrod & Ryan, 1975; Perel & Killinger, 1979; Folkerd *et al.*, 1982; Forney *et al.*, 1981; Deslypere *et al.*, 1985).

An important observation was that despite the large variation in levels of aromatase activity between different specimens of adipose tissue, activity was significantly higher in tissue obtained from women with breast cancer compared with that in adipose tissue from women with benign lesions. Others have failed to detect such a difference (Nimrod & Ryan, 1975; Perel & Killinger, 1979) but have studied very small numbers of patients. The larger study of Beranek and co-workers (1984) also reported no significant difference between cancer and control patients but employed less sensitive assay conditions utilising soluble rather than particulate fractions [the latter increase the sensitivity of aromatase assays (Nimrod & Ryan, 1975)]. Nevertheless aromatase activity was detected in 82% of cancer cases compared with only 50% of those with benign conditions suggesting a trend towards enhanced aromatase activity in adipose tissue from breast cancer patients.

There are two possible explanations for the presence of higher aromatase in breast adipose tissue from breast cancer patients, namely, either malignant tumours are capable of stimulating aromatase activity in surrounding tissues or regionally enhanced aromatase activity in adipose tissue produces a local environment which promotes malignant growth at that site.

With regard to the former possibility recent data suggest that growth factors enhance aromatase activity in peripheral tissues (McNeill *et al.*, 1986). These include factors which may be produced in a paracrine manner by tumours and preliminary results indicate that the addition of extracts from breast cancers to cultures of adipose tissue may stimulate oestrogen biosynthesis (Reed, 1986, personal communication).

Since oestrogens are heavily implicated in the promotion of breast cancer (Lacassagne, 1932; Symmers, 1968), local areas of enhanced oestrogen production might be expected to encourage development of tumour foci at these favourable sites. If this is the case one would expect a particular association between high aromatase activity in adipose tissue and oestrogen receptor positive tumours which are more likely to be oestrogen responsive. However such an association was not found in the present study.

As breast cancers in general have higher aromatase activity than adipose tissue (Abul-Hajj *et al.*, 1979; Perel *et al.*, 1980) the higher activity in adipose tissue from breast cancer patients could also be due to the presence of micrometastatic deposits of malignant cells within the adipose tissue. Whilst this possibility cannot be completely excluded, portions of fat adjacent to that used for the aromatase assay were not shown to be involved microscopically with cancer.

Although levels of aromatase activity were higher in fat from breast cancer patients, a large range of values was found in patients with and without malignant breast disease. It is clear therefore that other factors must influence the aromatase system. Statistically significant relationships were not observed between aromatase activity and age or menopausal status of the patients from whom the samples were obtained, although the highest levels of activitiy were observed in women aged between 40 and 55. This trend is similar to that noted in a study of abdominal adipose tissue (Folkerd *et al.*, 1982). However in view of the relative excess of pre-menopausal women in our benign group as compared with cancer patients and the reports of others that peripheral aromatase increases with age (Forney *et al.*, 1981; Cleland *et al.*, 1985), the data was reanalysed excluding post-menopausal women. The difference in aromatase activity between the cancer patients and the non-malignant group remained highly significant, so it is therefore extremely unlikely to be an age related phenomenon.

As we also failed to detect significant relationships between aromatase activity and various factors associated with excess risk of breast cancer such as height, weight, obesity, parity, age at menarche, age at first full term pregnancy, and family history of breast cancer, the differences between cancer and control patients are unlikely to be due to any excess of these risk factors in the cancer group. Factors which influence the inherent level of aromatase in breast adipose tissue still therefore require to be identified. However in view of the central role of oestrogen in both the aetiology and continued growth of endocrine sensitive tumours, the identification of such factors and the mechanisms by which they act may lead to the capacity to modify the local endocrine environment within the breast and could ultimately prove to be of value in the clinical managment of breast cancer.

We are grateful to Professor Sir Patrick Forrest for allowing us to study patients who were under his care, and for his advice and encouragement during the course of this study. We also wish to thank Dr T.J. Anderson and Dr J.J. Going for their cooperation in obtaining tissue samples. J.S. O'Neill was supported by a Hastilow Research scholarship from Edinburgh University Medical Faculty during this study.

### References

ABUL-HAJJ, Y.J., IVERSON, R. & KIANG, D.T. (1979). Aromatisation of androgens by human breast cancer. *Steroids*, **33**, 205.

BERANEK, P.A., FOLKERD, E.J., GILCHICK, M.W. & JAMES, V.H.T. (1984). 17 $\beta$ hydroxysteroid dehydrogenase and aromatase activity in breast fat from women with benign and malignant breast tumours. *Clin. Endocrinol.*, **20**, 205.

BERKOVITZ, G.D., FUJIMOTO, M., BROWN, T.R., BRODIE, A.M. & MIGEON, C.J. (1984). Aromatase activity in cultured human genital skin fibroblasts. *J. Clin. Endocrinol. Metab.*, **59**, 665.

BRADFORD, M.M. (1976). A rapid and sensitive method for the quantitation of microgram quantities of protein utilizing the principle of protein-dye binding. *Analyt. Biochem.*, **72**, 248.

CLELAND, W.H., MENDELSON, C.R. & SIMPSON, E.R. (1985). Effects of aging and obesity on aromatase activity of human adipose cells. *J. Clin. Endocrinol. Metab.*, **60**, 174.

DE OME, K.B., FAULKIN, L.J. & BERN, H.A. (1959). Development of mammary tumours from hyperplastic alveolar nodules transplanted into gland free mammary fat pads of female C3H mice. *Cancer Res.*, **19**, 515.

DESLYPERE, J.P., VERDONCK, L. & VERMEULEN, A. (1985). Fat tissue: A steroid reservoir and site of steroid metabolism. *J. Clin. Endocrinol. Metab.*, **61**, 564.

FOLKERD, E.J., REED, M.J. & JAMES, V.H.T. (1982). Oestrogen production in adipose tissue from normal women and women with endometrial cancer *in vitro*. *J. Steroid Biochem.*, **16**, 297.

FORNEY, J.P., MILEWICH, L., CHEN, G.T. & 4 others (1981). Aromatisation of androstenedione to estrone by human adipose tissue *in vitro*. Correlation with adipose tissue mass, age and endometrial neoplasia. *J. Clin. Endocrinol. Metab.*, **53**, 192.

FRIEDEN, E.H., PATKIN, J.K. & MILLS, M. (1968). Effects of follicle stimulating hormone (FSH) upon steroid aromatisation *in vitro*. *Proc. Soc. Exp. Biol. Med.*, **129**, 606.

GRODIN, J.M., SIITERI, P.K. & MACDONALD, P.C. (1973). Source of oestrogen production in the post-menopausal woman. *J. Clin. Endocrinol. Metab.*, **36**, 207.

LACASSAGNE, A. (1932). Apparition de cancers de la mamelle chez la souris male, soumise a des injections de folliculine. *C.R. Acad. Sci.* (Paris), **195**, 630.

McMAHON, B., COLE, P. & BROWN, J. (1973). Etiology of human breast cancer: A review. *J. Natl Cancer Inst.*, **50**, 21.

McNEILL, J.M., REED, M.J., LAI, L.C., NEWTON, C.J., GHILCHICK, M.W. & JAMES, V.H.T. (1986). The effect of EGF and TGF$\alpha$ on aromatase activity in cultured adipose tissue from pre- and post-menopausal women. *J. Endocrinol.*, **111**, (*Abstract 80*).

MILLER, W.R., FORREST, A.P.M. & HAMILTON, T. (1974). Steroid metabolism by human breast and rat mammary carcinomas. *Steroids*, **23**, 379.

NIMROD, A. & RYAN, K.J. (1975). Aromatisation of androstenedione by human abdominal and breast fat tissue. *J. Clin. Endocrinol. Metab.*, **40**, 367.

PEREL, E. & KILLINGER, D.W. (1979). The interconversion and aromatisation of androgens by human adipose tissue. *J. Steroid Biochem.*, **10**, 623.

PEREL, E., WILKINS, D. & KILLINGER, D.W. (1980). The conversion of androstenedione to oestrone, oestradiol and testosterone in breast tissue. *J. Steroid Biochem.*, **13**, 89.

PRESCHTEL, K. (1979). Cited in Barth, V. *Atlas of Diseases of the Breast*. Year Book Medical Publishers: Chicago, 1979, p. 11.

SYMMERS, W.St.C. (1968). Carcinoma of the breast in trans-sexual individuals after surgical and hormonal interference with primary and secondary sex characteristics. *Br. Med. J.*, **2**, 83.